# Supplementary material for: Evaluation of Amisulbrom Products for the Management of Clubroot of Canola (Brassica napus)
Source: Plants (Basel). 2023 Dec 21;13(1):28. doi: 10.3390/plants13010028 (PMC10780551; doi:10.3390/plants13010028)
Supplement: Supplementary file 1 [file plants-13-00028-s001.zip › plants-2728008-supplementary.pdf]

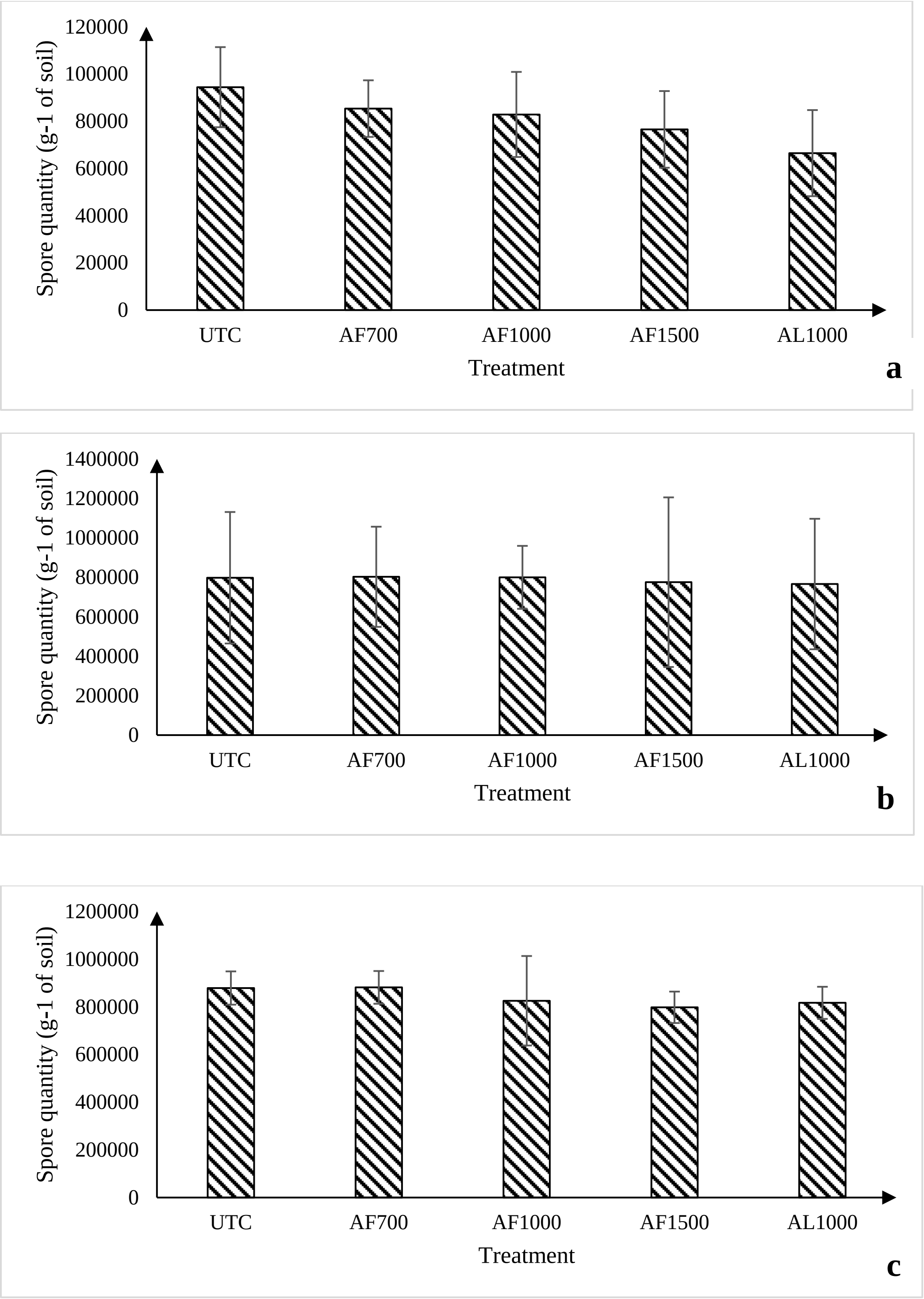

Figure S1. Effect of amisulbrom treatments on *Plasmodiophora brassicae* resting spore quantity in the soil under field conditions at Edmonton in 2019 (panel a), and at Edmonton Site 1 (panel b) and Site 2 in 2020 (panel c). UTC, untreated control; AF700,

granular amisulbrom at 700 g active ingredient (ai) ha^{-1}; AF1000, granular amisulbrom at 1000 g ai ha^{-1}; AF1500, granular amisulbrom at 1500 g ai ha^{-1}; AL1000, liquid amisulbrom at 1000 g ai ha^{-1}. Error bars indicate the standard deviation. No significant differences ($p < 0.05$) were detected for any of the treatments.
